# A Measure of the Parent-Team Alliance in Youth Residential Psychiatry: The Revised Short Working Alliance Inventory

**DOI:** 10.1007/s10566-015-9306-1

**Published:** 2015-02-13

**Authors:** Audri Lamers, Marc J. M. H. Delsing, Brigit M. van Widenfelt, Robert R. J. M. Vermeiren

**Affiliations:** 1 Department of Child and Adolescent Psychiatry, Curium-Leiden University Medical Center, Endegeesterstraatweg 27, 2342 AK Oegstgeest, The Netherlands; 2 Department of Psychology, Texas A&M University, College Station, TX USA; 3 Praktikon, Radboud University Nijmegen, Nijmegen, The Netherlands

**Keywords:** Therapeutic alliance, Psychometrics, Caregivers, Residential care, Youth psychiatry

## Abstract

**Background:**

The therapeutic alliance between multidisciplinary teams and parents within youth (semi) residential psychiatry is essential for the treatment process and forms a promising process variable for Routine Outcome Monitoring (ROM). No short evaluative instrument, however, is currently available to assess parent-team alliance.

**Objective:**

In this study, the Working Alliance Inventory-Short Version (WAV-12), a widely used alliance questionnaire, was adjusted to assess parent-team alliance from both a parent and team perspective within a youth residential setting. Psychometric properties, including factor structure and validity of the subscales, were explored.

**Methods:**

A sample of youth with mainly complex developmental disorders admitted to 11 inpatient and day patient units of a child and adolescent psychiatric institute participated in this study. The case manager involved with the youth and the primary caregiver of 87 youth completed the revised WAV-12 (WAV-12R).

**Results:**

The team version of the WAV-12R showed a good fit to the original conceptualized model, and distinguished Bond, Task and Goal scales. For the parents’ version an adjusted model with Insight, Bond and combined Task/Goal scales had the best fit. The reliability and validity of the scales were shown to be good.

**Conclusions:**

This paper presents preliminary evidence that the parent and treatment team versions of the WAV-12R are psychometrically sound for assessing parent-team alliance within youth (semi) residential psychiatry in the Netherlands. The team and parents’ versions of the WAV-12R are recommended instruments to complement outcome measures in ROM.

## Introduction

In youth mental health care, the therapeutic alliance of therapists with parents is regarded as a crucial component related to treatment success. The decision to seek treatment is rarely made by children or adolescents (hereafter youth) themselves. Instead, parents often lend their consent to treatment, provide transportation and may encourage youth to participate in treatment (Karver et al. 2005; Keeley et al. 2011; McLeod 2011; McLeod and Weisz 2005). Not surprisingly, the parental therapeutic alliance has been associated with treatment attendance and retention (Hawley and Weisz 2005; Kazdin et al. 2006). In addition, the more positive the therapeutic alliance between parents and therapists, the greater the therapeutic change in youth (Kazdin et al. 2005, 2006). Parents who are actively participating in treatment will more likely make changes that result in an environment conducive to positive outcomes of youth care (Karver et al. 2006). Even more, parents are often part of the therapeutic process, for example, in cases where they modify their parenting behavior by following parent training (Karver et al. 2005; McLeod and Weisz 2005). Stronger parental therapeutic alliances are related to more improved parenting skills (Kazdin et al. 2006). A meta-analysis of McLeod (2011) underscores the importance of the parental alliance in youth psychotherapy research, as their results indicated that the effect size of the alliance-outcome association was practically identical for the youth alliance and parental alliance.

Despite the valuable role of the parental therapeutic alliance in youth care, empirical research on parental alliance is scarce compared to the dozens of youth alliance studies (McLeod 2011) and hundreds of adult alliance studies (Horvath et al. 2011). A factor contributing to the lack of research in the youth field is the complexity of the construct of therapeutic alliance in youth mental health care compared to adult mental health care. There is no consensus on a general definition of therapeutic alliance in youth care research (Elvins and Green 2008; Zack et al. 2007). In adult psychotherapy, therapeutic alliance is generally conceptualized as consisting of three components: the bond or affective components of the relationship, agreement on the tasks or activities of the therapy, and shared agreement on the goals of the therapy (Bordin 1979). It has been argued that youth alliance may be viewed as a one-dimensional construct due to youth’s incapacity to discriminate between different components of the alliance (Elvins and Green 2008; Hogue et al. 2006; Roest et al. 2014; Shelef and Diamond 2008). Various youth alliance measures designed for use in youth psychotherapy have indicated one-factor solutions when subjected to factor analysis (Faw et al. 2005; Fjermestad et al. 2012). In contrast, for the parental alliance, the three components of Bordin (1979)—Bond, Task and Goal—might each play a role. Parents are often intensively involved in treatment planning, setting of treatment goals and conduct treatment tasks themselves (such as parent training). Unfortunately, parental alliance measures, until now, have not involved Bordin’s components (Accurso et al. 2013) or have neglected making a distinction between these components (Hawley and Garland 2008; Kazdin and Whitley 2006). Another factor complicating the conceptualization of the youth alliance construct in contrast to the adult alliance construct is that the youth alliance is not a mutual construction of a single relationship between a patient and therapist. Instead, relationship building occurs between youth, parents and therapists, who each have different roles within the therapeutic process. In most cases, at least two therapeutic alliances, youth and parents, are active during the treatment of the youth, which will have mutual influencing effects and shifts as treatment progresses. Therefore, it is striking that until now the alliance of youth and parents in youth care research has been measured with one therapist only (Accurso et al. 2013; Fjermestad et al. 2012; Hawley and Garland 2008). Especially in complex treatment settings, like youth psychiatry or residential youth care, a multidisciplinary treatment team is involved, instead of one therapist, in the treatment of youth. Classical alliance instruments might fail to capture relevant facets of the therapeutic alliance when more disciplines or a complete multidisciplinary team is involved (Blais 2004; Munder et al. 2010; Catty et al. 2012).

While the most severe and complex youth receive treatment in psychiatric (semi) residential settings, there has been minimal research in these settings to the parental alliance with the team. In this paper the term, (semi) residential psychiatry, is used for a multimodal treatment intervention, offered within an Institute for Child and Adolescent Psychiatry by a multi-Professional team, for youth with psychiatric disorders, which attend at least 3 days a week till a week long overnight stay. Three studies only report on the predictive value of the parental alliance, mostly assessed from the team’s perspective, for youth treatment results (Green et al. 2001, 2007; Kabuth et al. 2005). There is a lot to be gained if, in addition to the team’s information, information on the parental alliance would be collected from parents themselves in residential settings. A meta-analysis of McLeod (2011) showed that parent report of the youth alliance was more strongly linked to outcome than youth and observer reports. In earlier days, parents were often poorly involved during youth hospitalization; the idea was to break negative interaction chains between parents and youth (Verheij and Van Loon 1989). Nowadays, parents are often regarded as partners in the coordination of the treatment process for their children (Gross and Goldin 2008). Family involvement during residential treatment of youth has consistently been associated with improved treatment outcome (Hair 2005; Robst et al. 2013). Parents’ information on the therapeutic alliance might be crucial to the clinical therapeutic process of the treatment. Failure to establish a parental alliance may hinder treatment efforts, potentially increase the resistance of youth, and lead to premature termination of treatment. The “unsticking of stuck situations” of the residential team together with parents is often the way to therapeutic change in both the youth and the family (Gross and Goldin 2008). If parents and the team receive explicit information about the parental alliance, this might prevent stagnation and dropout during treatment. The parent-team alliance will—inevitably—come under strain at times during residential treatment (Green 2006); therefore instruments are needed that can regularly assess this process variable.

Since psychiatric (semi) residential treatment is one of the most intensive and costly treatments in youth care, psychometrically sound monitoring instruments could provide an opportunity to continuously evaluate and improve the quality of this treatment. Although the adult field has focused comprehensively on implementing Routine Outcome Monitoring (ROM), progress in the youth field lags behind (Bickman 2008). Creating routine measuring systems for youth is complex; developmental aspects of youth, different informants and contextual factors should be taken into account (Boer et al. 2012). Boer and colleagues (Boer et al. 2012) stressed the priority of developing ROM instruments related to parental factors in youth care. One factor worthwhile measuring in a ROM system is the therapeutic alliance with parents. When, in adult psychotherapy, feedback is given on therapeutic alliance as well as on outcomes within a ROM framework, clients are more likely to achieve a change of clinical significance (Whipple et al. 2003). Until now, in the youth research field the focus of ROM implementation has been primarily on outcome measures (Hall et al. 2013) rather than on process measures. Including an instrument assessing the parental alliance in youth care may essentially contribute to ROM.

Currently, no measure is available (a) to assess parental alliance routinely over time, (b) that is based on Bordin’s conceptualization of therapeutic alliance, (c) that measures parental alliance with a whole treatment team instead of one therapist only, (d) that is able to assess parents’ as well as the team members’ perspective on parental alliance, and (e) that is tailored to the complex setting of (semi) residential psychiatry. To address this gap in the literature and in the clinical practice of youth care, the Working Alliance Inventory—12 (WAV-12) (Stinckens et al. 2009), a Dutch–Flemish translation of the Working Alliance Inventory-Short version (WAI-S)(Tracey and Kokotovic 1989), was adjusted. The WAI-S questionnaire is originally derived from the Working Alliance Inventory (WAI) (Horvath and Greenberg 1986), which is the most commonly used therapeutic alliance measure in adult mental health research (Ross et al. 2011). The WAI is a 36-item paper-and-pencil self-report questionnaire, which captures the perception of the client and the therapist on Bordin’s (1979) three dimensions of the therapeutic alliance. Initially developed for and studied in outpatient adult settings, the WAI has also been adapted for use in other settings (Florsheim et al. 2000; Hintikka et al. 2006; Kazdin et al. 2005), and in other countries (Corbella and Botella 2004; Guédeney et al. 2005; Soygüt and Uluc 2009; Vertommen and Vervaeke 1996). The measure aims to be nonspecific to either treatment technique or theory (Horvath and Greenberg 1989; Martin et al. 2000). A shortened 12-item version of the instrument, the WAI-S, was developed by selecting the four highest loading items of each of the three subscales—Goal, Task and Bond (Tracey and Kokotovic 1989). Subsequent factor analyses of the WAI-S with different adult populations found support for either one-, two- (Bond factor and a combined Goal/Task factor referred to as Work factor) or three-factor (Bond, Goal and Task factor) models (Andrusyna et al. 2001; Corbière et al. 2006; Horvath and Greenberg 1989; Horvath and Luborsky 1993; Tracey and Kokotovic 1989). Belgian colleagues translated the WAI-S to Flemish–Dutch (WAV-12) to measure the therapeutic alliance in adult psychotherapy and found support for the reliability of the three different subscales, with high Cronbach’s alphas: client version (0.92), therapist version (0.94), with subscale alphas ranging from 0.81 to 0.93 (Vertommen and Vervaeke 1996).

An adjusted version of the Dutch–Flemish WAV-12 to assess the parent-team alliance in youth residential psychiatry may be a useful, low burden, and an accurate instrument to be part of a routine monitoring system. Therefore, adaptations are needed of the two versions of the WAV-12 to the youth psychiatric residential setting, the target group, and the Dutch culture. The client version was transformed into a parent version and the therapist version into a team version. Next, the psychometric properties of the two adapted versions of the WAV-12 were examined in a sample of youth, mostly diagnosed with developmental disorders, who were admitted to residential units (mostly day treatment) of a youth psychiatric institute. In the Netherlands, semi-residential settings generally involve a large number of youth with developmental disorders, as also reported by De Jonge et al. (2003). As the parental alliance in (semi) residential psychiatry is as yet not clearly defined as a concept, the present study aims to take a closer look at the underlying structure of the adjusted WAV-12 versions. Firstly, it was expected that factor analysis would reveal that Bordin’s (1979) three components found in adult psychotherapeutic settings would also apply to parental alliance. It is important to identify the different components that account for team members’ and parents’ view of parental alliance because they each might be associated differently with outcome variables of (semi) residential treatment. Secondly, based on the strong psychometric foundation of the WAI-S (Busseri and Tyler 2003) and on previous findings regarding the WAV-12 (Stinckens et al. 2009), it was expected that both the internal consistency and concurrent validity of the subscales would be moderate to good. Given the context of a psychiatric residential setting with a large number of youth with severe and complex developmental disorders, the validation of the adapted WAV-12 is a necessity. In sum, the aim of the present study was to adjust the WAV-12 to the setting of youth residential psychiatry in the Netherlands and to evaluate its psychometric properties.

## Methods

### Setting

This study included youth who were admitted to one of the 11 (semi) residential psychiatric units of an academic child psychiatric treatment center in the Netherlands. The units are located in two cities in the western part of the Netherlands and each provides treatment for seven to eight youth. These youth, ranging in age from 5 to 18 years, become admitted when experiencing severe psychiatric problems in combination with impaired personal, family and/or school functioning. The only exclusion criterion is an IQ less than 70. Referral sources include the institute’s outpatient setting, general practitioners and youth health-care centers. Youth attend semi-residential treatment for at least three but usually 5 days a week, for 8 h a day. In inpatient settings, the youth stay overnight for at least 5 days a week. A multidisciplinary approach is applied, which consists of the therapeutic milieu on the ward, parent counseling/training, educative therapy, psychomotor therapy and creative therapy. Treatment includes a highly structured day schedule in which social settings, such as school and sports, are integrated. A child psychiatrist or clinical psychologist is connected to the youth as a case manager and has overall responsibility for the treatment of the youth. Other clinicians involved are group care workers, creative therapists, psychomotor therapists and parent counselors. The primary goal of (semi) residential treatment is reducing psychiatric symptoms and improving youths’ quality of life and well-being. Treatment goals are tailor-made and can include diagnostics by means of intensive observation, reduction of anxiety symptoms, increase in adaptability, improvement of peer relations and increase in self-confidence.

### Participants

Primary caregivers and case managers of 93 youth were involved as participants. Case managers, two psychiatrists and three clinical psychologists, had more than five years of experience in child and adolescent psychiatry. The youth were admitted between June 2011 and December 2012 to 11 day and inpatient units. One referred client in this sample was excluded due to insufficient knowledge of the Dutch language. All but five of the clients gave permission for the use of their ROM data for research purposes. Analysis of the reasons why some participants did not respond after having given permission revealed that missing data were due to factors like the unforeseen fusion of two units, planned discharge and unavailability of case managers at the moment of data collection. In the case of 87 youth, data were available from one or two informants: 80 (response 86 %) from case managers and 73 (response 78 %) from primary caregivers (mostly mothers, but also two fathers). The 87 youth (79 % male) participating in the study ranged in age from 5.6 to 17.3 years, with a mean age of 10.3 years (SD = 3.2), of whom 17 were treated as inpatients and 70 received day treatment. The majority (71 %) of these youth received a primary DSM IV classification within the autistic spectrum, as assessed by the case manager after 3 months of treatment, 8 % were classified as having a behavioral disorder, 6 % as having anxiety/emotional disorders, while 15 % were classified otherwise. Of these youth, 54 grew up in complete families, 17 in one-parent families, seven had co-parents, seven grew up with a mother and a stepparent and two with foster parents.

### Procedures

The study was submitted to the medical ethical board of the University Medical Center in Leiden and approved as being in accordance with the medical ethical law in the Netherlands. Participants were informed before intake that ROM is part of the clinical setting’s general policy to monitor treatment outcome and that questionnaire data were to be used in an anonymous form for research purposes, as done by De Beurs and colleagues in adult psychiatry in the Netherlands (De Beurs et al. 2011). For 46 of the 93 youth, data were collected around the fourth month of treatment and for the remaining 47 at a random point in time during treatment. The youths’ questionnaires were completed around the same time by their caregiver and case manager, mostly online, but also on paper, and returned in a sealed envelope.

### Adaptation and Pilot Testing of the WAV-12R (Treatment Team and Parent Version)

After receiving approval from the Flemish authors, the versions of the WAV-12 (Stinckens et al. 2009) were adapted to measure the parent-team therapeutic alliance from two perspectives in a Dutch youth residential psychiatric setting. A team of three clinical psychologists and researchers made adjustments in multiple steps to adjust the Dutch versions of the Belgian WAV-12, thereby taking into account the specific setting in which the questionnaire was to be used and the Dutch culture and language. The most important adjustments to the WAV-12 were: (a) the expression ‘therapist’ was replaced by ‘treatment team’; (b) the term ‘client’ was changed to ‘parents of the child who is in treatment’; (c) the blank line that needed to represent the name of the therapist or client in the original questionnaire was replaced by ‘treatment team’ or ‘parent’; (d) the terms ‘sessions’ and ‘therapy’ were changed to ‘treatment’; and (e) for reasons of clarification, ‘we’ was replaced by ‘the treatment team and I.’ Next, a pilot test was done with the Dutch WAV-12R in a sample of 20 youth with case managers and the primary caregiver as informants. In a form attached to the questionnaire, the informants were asked to give feedback on three aspects of the Dutch WAV-12 versions. They were asked to respond to the content of the items, the rating options and the appropriateness of the Dutch formulation of the items. Based on the findings, two Belgian terms were replaced by more commonly used words in the Netherlands. One of these terms involves the fourth response option of the five-point Likert scale, ranging from 1: ‘rarely or never’ to 5: ‘always.’ The other one refers to one Belgian expression that is seldom used in the Netherlands. The final versions of the WAV-12R (see Box 1 for English translation of the items) were reported back to the Belgian authors of the WAV-12 versions. Back translation was not deemed necessary given the shared language of Belgium and the Netherlands. The ‘Bond’ scale consists of items 3, 5, 7 and 9, the ‘Goal’ scale of items 1, 4, 6 and 11 and the ‘Task’ scale of items 2, 8, 10 and 12. Both the team and parent versions of the Dutch WAV-12R have the same format, involving 12 items with slightly different formulations.

**Box 1:** English translation of the items for the two versions of the WAV-12R

*Questions in the treatment team version of the WAV*-*12R*
1. One result of this treatment is that it is clearer for parents how they and their child could change
2. Parents and I have confidence in the usefulness of our current activities in the treatment
3. I believe that parents like me
4. Parents and I worked together to determine treatment goals
5. Parents and I respect each other
6. Parents and I work on treatment goals we all agreed upon
7. I appreciate parents as persons
8. Parents and I agree about what is important to work on
9. I respect parents, even if they do things I don’t approve of
10. I am confident that the things we do in treatment will help parents to achieve the changes they want for their child and family
11. The parents and I have formed a good understanding of the kind of changes that would be good for their child and them
12. Parents believe that the way of working on the problems of their child is the right way
*Questions in the caregiver version of the WAV*-*12R*
1. One result of this treatment is that it is clearer for me how my child can change
2. What I do in this treatment gives me more insight into my child’s problems
3. I believe the treatment team likes me
4. The treatment team and I work together in determining the treatment goals
5. The treatment team and I respect each other
6. The treatment team and I work on treatment goals we all agreed upon
7. I feel appreciated by the treatment team
8. The treatment team and I agree about what is important for us to work on
9. I feel the treatment team cares for us, even if we do things they disapprove of
10. I think that my contribution to this treatment will help me and my child to achieve the changes we want
11. The treatment team and I have formed a clear understanding of the kind of changes that would be good for us
12. I believe that the way we work on the problems is the right way

### Measures Used for the Evaluation of Concurrent Validity

To investigate the concurrent validity of the treatment team version of the WAV-12R, the Family Engagement Questionnaire (FEQ) (Elvins and Green 2008; Kroll and Green 1997) was used. The FEQ is designed to measure the youth and parental alliance in youth inpatient settings and originally consists of 18 items (Kroll and Green 1997). The FEQ was translated into Dutch by Lamers and Van Widenfelt (2014). In addition to two ‘Youth Alliance’ scales, the Dutch version of the FEQ consists of a ‘Parental Alliance’ scale. The latter was used to find proof for the concurrent validity of the WAV-12R. The ‘Parental Alliance’ scale, which had a Cronbach’s alpha of 0.69 in the current sample, refers to the involvement and confidence of parents in the treatment. The scale consists of four items that were rated on four-point Likert scales ranging from ‘most of the time’ to ‘almost never’. The ‘Parental Alliance’ scale was presented to the case manager involved with the particular youth.

The Dutch version of the Empathy and Understanding Questionnaire (Green 1996) was used to evaluate the concurrent validity of the parent version of the WAV-12R. The EUQ was developed by the same research team in the United Kingdom, and translated by the same team in the Netherlands, as the FEQ. The EUQ covers understanding of the treatment rationale, experience of empathy from the staff, perceived accuracy of empathy and subjects’ sense of collaboration within the treatment process. The EUQ, which had a Cronbach’s alpha of 0.77 in the present sample, consists of six items that were rated on four-point Likert scales with predefined answer categories. The youth’s primary caregiver filled in the EUQ and the total scale score was used in this study to evaluate the concurrent validity of the parent version of the WAV-12R.

### Statistical Analyses

A series of confirmatory factor analyses (CFAs) was conducted to test whether either a one-, two- (Bond factor and a combined Goal/Task factor referred to as Work factor), or three-factor (Bond, Goal and Task factors) model showed the best fit to the data for both the parent and team version of the WAV-12R. The CFAs were performed using Mplus 5.1 (Muthén and Muthén 1998). A full-information maximum likelihood (FIML) estimator with robust standard errors was used, implemented as MLR in Mplus 5.1, to make use of all the available data. The COMPLEX module implemented in Mplus 5.1 was used to account for nonindependence of observations due to cluster sampling (case managers reported with regard to more than one parent). The assessment of model fit involves an inspection of the factor loadings as well as an examination of ‘fit statistics.’ Each fit statistic provides information about the degree to which the model fits the observed data. As the current sample is somewhat small for CFA, fit statistics were chosen that appear to remain accurate even in smaller samples. Moreover, the maximum likelihood estimation procedure, used in this study, requires somewhat smaller sample sizes (Kline 2005). The Comparative Fit Index (CFI) (Bentler 1995), the Root Mean Square Error of Approximation (RMSEA) (Steiger 1990) and the Standardized Root Mean Square Residual (SRMR) (Bentler 1995) were used to evaluate model fit. According to generally accepted cutoff values, CFI values >0.90 represent an acceptable fit and >0.95 represent a good fit; RMSEA and SRMR values between 0.05 and 0.08 suggest an acceptable fit, and >0.10 a poor fit, whereas values <0.05 indicate a good fit (Hu and Bentler 1999). If necessary, adjustments were made to the models on the basis of the modification indices in order to improve the fit (Bacher 1987). Subsequent analyses were performed using SPSS 19.0. Internal consistency reliability was assessed for each subscale using Cronbach alphas. Reliability coefficients <0.60 are considered insufficient, 0.60 to 0.69 marginal, 0.70 to 0.79 acceptable, 0.80 to 0.89 good and 0.90 or higher excellent (Barker et al. 1994). Concurrent validity was assessed using Pearson correlations; coefficients ≥0.50 are considered strong (Cohen 1988).

## Results

### Factor Analyses Team Version

With regard to the team version of the WAV-12R, the fit statistics for the three models tested for case managers’ reports are presented in the upper part of Table 1. The one-factor model, as well as the two-factor model, had an acceptable fit according to the CFI and SRMR. According to the RMSEA, however, the fit was poor for both models. The three-factor model, with a ‘Bond,’ ‘Goal’ and ‘Task’ factor, revealed an acceptable fit according to the SRMR value and a good fit according to the CFI value. The RMSEA value is just above the 0.08 cutoff value.

**Table 1:** Goodness-of-fit indices for the different models of both the treatment team and parent version of the WAV-12R

Model	*Df*	S–Bχ^2^	CFI	RMSEA	SRMR	ΔS–Bχ^2^
WAV-12R Team version (*n* = 80)						
1-factor (General alliance)	54	113.66	0.93	0.12	0.06	
2-factor (Bond, Work)	53	98.74	0.94	0.10	0.06	38.39**
3-factor (Bond, Task, Goal)	51	81.06	0.96	0.09	0.06	7.38*
WAV-12R caregiver version (*n* = 73)						
1-factor (General alliance)	54	144.52	0.81	0.15	0.09	
2-factor (Bond, Working)	53	101.69	0.90	0.11	0.07	102.09**
3-factor (Bond, Task and Goal)	51	102.51	0.89	0.12	0.07	0.38
3-factor (Insight, Bond, Work)	52	71.71	0.96	0.08	0.06	22.57**

*S–Bχ* ^*2*^ Satorra–Bentler Chi square, *CFI* Comparative Fit Index, *RMSEA* root mean square error of approximation, *SRMR* standardized root mean square residual. ΔS–Bχ^2^ = Satorra–Bentler Chi square difference test

* Significant at the 0.05 level; ** significant at the 0.01 level

**Fig. 1:**
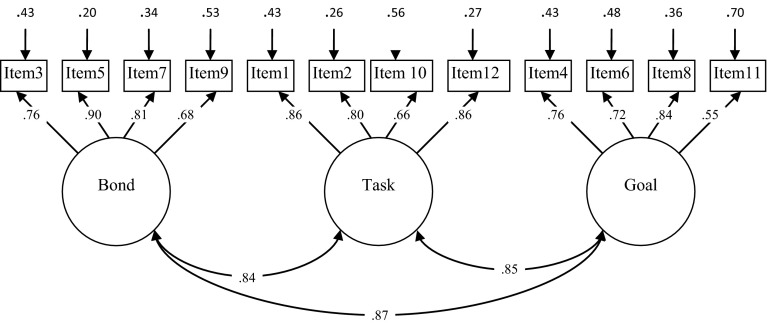
Factor loadings, intercorrelations and error variance of the team version of the WAV-12R for a 3-factor model (N = 80)

Figure 1 shows the standardized parameter estimates for the three-factor model. The factor loadings of the items on the supposed underlying factor were all significant. Although intercorrelations between factors were strong, Chi square difference tests revealed that the three-factor solution fit significantly better than both the one- and two-factor model, suggesting that the factors reflect different constructs.

### Factor Analyses Parent Version

**Fig. 2:**
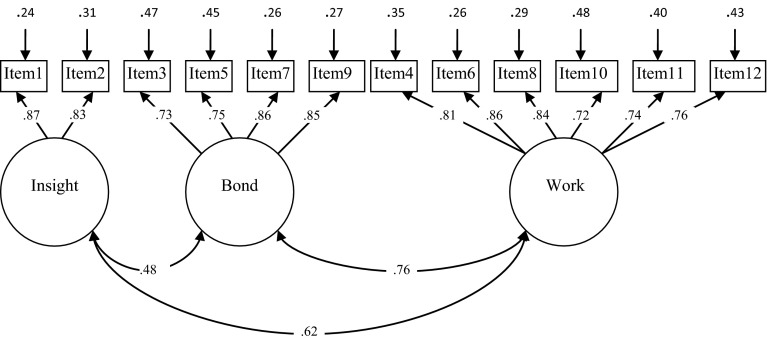
Factor loadings, intercorrelations and error variance of the WAV-12R (caregiver version) for an adjusted 3-factor model (N = 73)

As can be seen in the lower part of Table 1, the two-factor model of the caregiver version of the WAV-12R, with a ‘Bond’ and ‘Work’ (combined Goal/Task) factor, revealed an acceptable fit according to the CFI and SRMR values. However, the RMSEA value of 0.11 indicates a poor fit. The one-factor model and the three-factor model also showed a poor fit as indicated by the CFI and RMSEA values. Inspection of the modification indices of the two-factor model suggested a strong correlation between the first two items of the questionnaire. These items are: “One result of this treatment is that it is clearer for me how my child can change” and “What I do in this treatment gives me more insight into my child’s problems.” Both items seem to capture a separate factor referring to the insight of the caregiver. Accordingly, an adjusted model was tested distinguishing three factors: the new factor labeled ‘Insight’ (items 1 and 2), ‘Bond’ (items 3, 5, 7 and 9) and ‘Work’ (items 4, 6, 8, 10, 11 and 12) alliance. This alternative model revealed an acceptable fit according to the RMSEA and SRMR values and a good fit according to the CFI value. A Chi square difference test showed that the final model fit the data significantly better than the two-factor model. Since our final three-factor model and Bordin’s three-factor model are not nested, a Chi square difference test to compare both models cannot be performed. However, the higher CFI value and lower RMSEA and SRMR values suggest that the adjusted three-factor model has a much closer fit to the data than Bordin’s original model. In Fig. 2, item loadings (all significant and >0.72), correlations between factors and error variances are presented. The correlations between factors indicate that the factors reflect relatively independent dimensions of the therapeutic alliance.

### Internal Reliability and Concurrent Validity of the Subscales

Reliability coefficients of each subscale, ranging from 0.78 to 0.97, were acceptable to excellent, as can be seen in the upper part of Table 2. To evaluate the concurrent validity of the different subscales of the two versions of the WAV-12R, Pearson’s product moment correlations were computed (see lower part of Table 2). For the caregiver version the scores on the subscales and total scale of the primary caregiver on the WAV-12R were correlated with their total score on the Empathy and Understanding Questionnaire. These correlations ranged from 0.50 to 0.78, providing support for the concurrent validity of the instrument. The correlations between case managers’ reports on the WAV-12R and the ‘Parental Alliance’ scale of the Family Engagement Questionnaire were also strong for the ‘Bond,’ ‘Goal’ and ‘Total’ scale and ranged from 0.53 to 0.57. The ‘Task’ scale of the treatment team version had a correlation of 0.48 with the ‘Parental Alliance’ scale of the FEQ.

**Table 2:** Cronbach’s alpha’s of the Subscales of the two versions of the WAV-12R and Pearson correlations with related alliance questionnaires

	WAV-12R team version (*n* = 78)				WAV-12R parent version (*n* = 67)			
	Bond	Task	Goal	Total	Bond	Work	Insight	Total
Internal reliability	0.97	0.87	0.78	0.93	0.87	0.92	0.84	0.93
Concurrent validity								
Total score, EUQ	–	–	–	–	0.50**	0.78**	0.54**	0.75**
Parental scale, FEQ	0.57**	0.48**	0.53**	0.56**	–	–	–	–

*EUQ* Empathy and Understanding Questionnaire completed by caregiver, *FEQ* Family Engagement Questionnaire completed by case manager

** Significant at the 0.01 level

## Discussion

The main purpose of the current study was to advance the literature on the conceptualization and measurement of the parental therapeutic alliance in complex youth treatment settings, guided by the belief that the parental alliance is an important variable for ROM in youth care. At present, no measure is available for routinely measuring the parent-team alliance, which distinguishes between Bond, Task and Goal components and includes team members’ as well as parents’ perspective. This study reports on the psychometric properties of a short measure of the parent-team therapeutic alliance in a sample of youth with predominantly complex developmental disorders in youth (semi) residential psychiatry. The WAV-12R was developed by adjusting the WAI-S, which is the most used short alliance measure for adult psychotherapy. The main findings were: (1) that for the case managers’ version of the WAV-12R, Bordin’s (1979) original model distinguishing a Bond, Task and Goal factor showed an acceptable fit to the data; (2) that for the caregivers’ version of the WAV-12R, an adjusted model with an Insight, Bond and Work (combined Task/Goal) factor showed a good fit to the data; (3) that the resulting scales of both revised versions of the WAV-12R showed strong internal consistencies and concurrent validity. These findings justify the use of the WAV-12R for routine outcome measurement in (semi) residential psychiatry for complex developmentally disturbed youths. Hence, the treatment team may use the WAV-12R as an instrument to monitor the parent-team therapeutic alliance with the advantage of gathering the team members’ as well as the parents’ perspective. Parents will most likely feel more strongly involved in the treatment of their child when the team explicitly asks for their information on the therapeutic alliance. The fact that the WAV-12R distinguishes between different aspects of the parental alliance enables assessment of how these aspects change over time and are differentially related to outcome. For example, the Bond alliance may be more important at the start, while Goal and Task alliances may be more important during the middle and at the end of treatment or the other way around. This makes the WAV-12R a valuable tool to enrich the current scarce literature on the parental alliance in (semi) residential youth treatment settings.

Evidence for the construct validity of the two versions of the WAV-12R was found by means of confirmatory factor analysis. As expected, case managers’ ratings on the WAV-12R produced an acceptable fit to the dimensions of the therapeutic alliance proposed by Bordin (1979): Bond, Goal and Task. This finding is in line with research on the WAI-S in inpatient adult mental health, which also confirmed a three-factor model (Munder et al. 2010). These findings contribute to a more specific conceptualization of the parental alliance construct in the youth care literature. Although youth alliance has been conceptualized as a one-dimensional construct (Elvins and Green 2008), the same components valid for adult alliance seem to apply to parental alliance. Evolution of parents’ involvement and engagement in the (semi) residential treatment of their youth may lead to the growth of the partnership relationship between them and the team. Treatment teams promote parent participation in active problem solving and joint decision making about the care of their youth (Fowler et al. 2012). Case managers evaluate the treatment plan together with youth and parents and mutually design the tasks and goals of the youths’ treatment.

With regard to primary caregivers, support was found for an adjusted model of parental alliance, distinguishing an Insight, Bond and Work (combined Goal/Task) factor. The differentiation between the Bond factor on the one hand, and the combined Goal and Task factor on the other, is in accordance with other studies investigating the factor structure of the WAI (Andrusyna et al. 2001) and WAI-S (Andrusyna et al. 2001; Ross et al. 2011) in adult psychotherapy. The factor “Insight” found in this study for caregivers’ reports, involving parents’ insight into the problems and the possibility of change, is most likely specifically for the population of severe and complex mental health disorders. Remarkably, insight has also been mentioned as an important construct related to the therapeutic alliance in the treatment of adults with severe mental illnesses (McCabe and Priebe 2004). How a person makes sense of his or her experiences is fundamental to therapeutic interaction (McCabe and Quayle 2002). When clients have a different explanatory model than their therapist about a disorder, this has an impact on clients’ adherence to the treatment (Nock and Ferriter 2005). A shared explanatory model of illness promotes a positive collaboration and communication between clinician and patient (Bhui and Bhugra 2002; Callan and Littlewood 1998). One of the reasons for referral of youth to residential treatment is that the problems of the youth and their families are so complex that diagnoses remain unclear. For parents of youth in (semi) residential psychiatry, an accurate awareness of the problems and optimism about change might be an important facilitator for a positive therapeutic alliance. The ‘Insight’ scale of the WAV-12R opens up opportunities for researchers and care providers to examine its relation to youth residential treatment outcomes.

Within the context of residential psychiatry, the concept of the parental alliance may differ somewhat across informants. Until now, research on the Working Alliance Inventory (Short Version) has found no differences between the factor structures of different raters (Ross et al. 2011). Taken together, these results support the perspective of Boer et al. (2012) that in youth mental health care different informants should be involved when measuring process or outcome variables. Highlighted is the need to examine the generalizability of the factor structure of routine measuring instruments to establish their measurement invariance across different informant perspectives.

Next, it was expected that the results regarding the internal consistency reliability and the concurrent validity of the subscales of both the treatment team and parent version of the WAV-12R would be consistent with earlier research with the WAI-S and WAV-12. Results suggest that the scale constructs of the two versions of the WAV-12R can be reliably assessed by means of these questionnaires. Reliability coefficients were as high as those reported for the WAV-12 (Vertommen and Vervaeke 1996) and the WAI-S (Tracey and Kokotovic 1989). Significant correlations between the subscales and total scale of the team version of the WAV-12R and the Family Engagement Questionnaire provide initial support for the measure’s concurrent validity. Similarly, the scores on the subscales and total scale of the parent version of the WAV-12R significantly correlated with scores on the Empathy and Understanding questionnaire. The subscale ‘Parental Alliance’ of the FEQ seemed to be covered by items related to ‘Bond’ aspects rather than to ‘Task’ and ‘Goal’ aspects. Most likely as a result, the correlations for these last two scales were somewhat lower. In sum, most indicators of psychometric quality suggest that the parent and treatment team versions of the WAV-12R perform well as measures of the parent-team alliance for youth with severe developmental disorders in (semi) residential psychiatry.

## Limitations

This study was conducted in a challenging and complex treatment setting resulting in several limitations. Firstly, the present sample was smaller than is typically recommended for Confirmative Factor Analyses (Fan et al. 1999; Kline 2005). Although the fit statistics reported here are thought to minimize the statistical effect of a smaller sample, it is possible that this may have affected the results. Secondly, we do not know to what extent our findings can be generalized to other patient groups that differ in age, informants, treatment contents and psychopathologies. For example, most youth who participated in the current study had an autism spectrum disorder, with high rates of comorbidity (i.e. behavior and anxiety disorders). Therefore, replication of this study with different subgroups of residential youth is recommended. Thirdly, future studies could further investigate the concurrent validity of the parental alliance construct in youth (semi) residential psychiatry by distinguishing the different components, Bond, Goal and Task. Finally, it is unclear whether, for example, the internal structure and validity of the WAV-12R can be replicated when caregivers and case managers complete the WAV-12R with the goal of providing feedback. The effect of ROM is especially meaningful when feedback is given to the participants. Although Summers and Barber recommended in 2003 that psychiatry residency programs consider measuring therapeutic alliance as a tool for feedback, until now this has not been effectuated (Summers and Barber 2003). The WAV-12R might be a valuable clinical tool for building stronger parental alliances. For future research it is recommended that the sensitivity of the WAV-12R versions to over-time changes in therapeutic alliance should be investigated, and that the effect of providing feedback about the therapeutic parent-team alliance on treatment outcome should be explored.

## Conclusions

Instruments that enable routine assessment of the parent-team therapeutic alliance in youth residential psychiatry are necessary for research purposes, and vital for sound clinical practice. The psychometric properties of the parent and treatment team versions of the Dutch WAV-12R in youth residential psychiatry were supported in this study. The ability to measure the parental alliance in residential youth psychiatric settings at multiple time points will help theory and treatment development as well as the implementation of ROM. This in turn may lead to improvement of important aspects of youth treatment in this specific setting. Given the widely acknowledged importance of therapeutic alliance, the parent-team therapeutic alliance in a youth residential setting deserves more empirical and clinical attention.
